# Pseudogenes as Weaknesses of ACTB (Actb) and GAPDH (Gapdh) Used as Reference Genes in Reverse Transcription and Polymerase Chain Reactions

**DOI:** 10.1371/journal.pone.0041659

**Published:** 2012-08-22

**Authors:** Yuan Sun, Yan Li, Dianzhong Luo, D. Joshua Liao

**Affiliations:** 1 Hormel Institute, University of Minnesota, Austin, Minnesota, United States of America; 2 Department of Pathology, Guangxi Medical University, Nanning, Guangxi, People's Republic of China; Wayne State University, United States of America

## Abstract

The genes encoding β-actin (ACTB in human or Actb in mouse) and glyceraldehyde-3-phosphate dehydrogenase (GAPDH in human or Gapdh in mouse) are the two most commonly used references for sample normalization in determination of the mRNA level of interested genes by reverse transcription (RT) and ensuing polymerase chain reactions (PCR). In this study, bioinformatic analyses revealed that the ACTB, Actb, GAPDH and Gapdh had 64, 69, 67 and 197 pseudogenes (PGs), respectively, in the corresponding genome. Most of these PGs are intronless and similar in size to the authentic mRNA. Alignment of several PGs of these genes with the corresponding mRNA reveals that they are highly homologous. In contrast, the hypoxanthine phosphoribosyltransferase-1 gene (HPRT1 in human or Hprt in mouse) only had 3 or 1 PG, respectively, and the mRNA has unique regions for primer design. PCR with cDNA or genomic DNA (gDNA) as templates revealed that our HPRT1, Hprt and GAPDH primers were specific, whereas our ACTB and Actb primers were not specific enough both vertically (within the cDNA) and horizontally (compared cDNA with gDNA). No primers could be designed for the Gapdh that would not mis-prime PGs. Since most of the genome is transcribed, we suggest to peers to forgo ACTB (Actb) and GAPDH (Dapdh) as references in RT-PCR and, if there is no surrogate, to use our primers with extra caution. We also propose a standard operation procedure in which design of primers for RT-PCR starts from avoiding mis-priming PGs and all primers need be tested for specificity with both cDNA and gDNA.

## Introduction

Determination of mRNA level of an interested gene in eukaryotic cells often involves conversion of the mRNA to cDNA by reverse transcription (RT), followed by polymerase chain reactions (PCR). This RT-PCR approach is much more sensitive than other methods such as Northern blot, because PCR amplifies the cDNA in an exponential manner. So often, the RT-PCR products need to be compared between two, or among more, samples to determine whether some sample(s) have a different mRNA level of the interested gene from the others [1]. In this case, a reference gene is needed for sample normalization, i.e. for assessing that an equal amount of the RT products from all the samples is used as template in the PCR. Because RT-PCR is so sensitive that it can detect the mRNA level even in a single cell, variation in the expression level of the reference gene needs to be tightly controlled, otherwise a bias may be produced. Ideally, expression of the reference gene should be constant in all situations and should be refractory to all the changes in the experimental conditions. At least, its expression should not be changed by the to-be-studied situation.

There has been a long list of genes that have been used as references in RT-PCR [2]–[6], of which the genes encoding β-actin (ACTB in human or Actb in mouse, according to the NCBI nomenclature of genes) and glyceraldehyde-3-phosphate dehydrogenase (GAPDH in human and Gapdh in mouse) are the two most frequently used ones [1]. The hypoxanthine phosphoribosyltransferase-1 (HPRT1 in human and Hprt in mouse) is also used often [5], [7], [8]. The reason for having so many reference genes is because none of them really meets the above mentioned ideal criteria, and therefore researchers have to select different ones according to their to-be-studied situations [9]–[11]. The weaknesses of most reference genes have been discussed in the literature and pertain mainly to the stability or variation of their expression in different situations [12]–[14], with only very few concerning the influence of pseudogene (PG) in their fidelity as references [15]–[17]. ACTB, Actb, GAPDH or Gapdh had been used as references in RT-PCR long before the human and mouse genomes were fully sequenced. Although individual PGs of these genes in the human and mouse were reported a long time ago, these genes continue serving as references, because some peers do not realize that these genes have PGs while many others consider that most PGs are not transcribed, which actually has been a generally accepted concept for a long time.

According to a report from the human genome project, about 1.1% of the human genomic DNA belongs to exons while 24% belongs to introns, together about a quarter of the genome owned by the protein coding genes [18]. However, the majority of the remaining three quarters is not junk and is actually also transcribed at least in some cell types or at some times [19]. After sequencing the RNA transcripts from about 1% of the human genome, the ENCODE pilot project reports that 93% of the bases in this 1% of the genome are transcribed [20]. The human genome contains only about 20,000 protein coding genes but about 19,000 PGs, although probably less than 20% of the PGs are commonly transcribed [21]. Like most other non-coding RNAs, many PG transcripts may be functional, such as in regulation of the expression of their parental genes [21]–[23]. There are even further lessons that in some situations processed PGs are transcriptionally activated but are mistaken as the turn-on of the authentic genes that are actually inactive [24]–[26]. These facts actually arouse in us a concern as to whether genetic knockout of one gene would change its PG expression or, after some latent period of time, even trigger expression of some of its PGs that are otherwise silent in some tissues or cell types, since reports on gene-knockout animals hardly address this aspect. In short, these latest advances in RNA biology are revolutionary to biomedical science as they not only challenge the definition of “gene” [27] and many other fundamental concepts in our mind but also require us to reevaluate some experimental methodologies. As an example, in this report we provide bioinformatic data showing that ACTB (Actb) and GAPDH (Gapdh) have many PGs in the human and mouse genomes, which may affect the fidelity of these genes as references for RT-PCR.

## Materials and Methods

In the database of National Center for Biotechnology Information (NCBI) of the United States, the mRNA sequence of all genes is presented as DNA sequence, i.e. uracil (U) is replaced by thymine (T). According to the nomenclature of the NCBI, the name of human genes should be fully capitalized whereas the name of mouse genes should be capitalized only the first letter. We pulled out the mRNA sequences of ACTB, Actb, GAPDH, Gapdh, HPRT1 and Hprt from the NCBI database; the gene identification (gi) number and mRNA access number were provided in Fig. S1, prior to the corresponding sequence. PGs were identified using online software and databases as indicated. An online software (http://biotools.umassmed.edu/bioapps/primer3www.cgi) was used for primer design. Insilico PCR was performed with two different online software packages (http://insilico.ehu.es/PCR/ and http://genome.csdb.cn/cgi-bin/hgPcr).

The cell lines from which data were presented include GI101A human breast cancer cell line as well as Panc-1, Panc-28, Coolo357 and L3.5pL human pancreatic cancer cell lines; all these cell lines are well documented in the literature. E6E7st non-transformed and E6E7st/ras transformed human pancreatic ductal epithelial cell lines were provided by Dr. Paul Campbell [28]. M8 mouse pancreatic [29] and ND5 mouse breast [30] cancer cell lines were established by us previously. All cell lines were cultured with DMEM containing 5% bovine serum and were harvested when the cells reached about 70% confluence. Isolation of total RNA from the cells was performed using Trizol (Invitrogen, Cat. 15596-026; www.invitrogen.com), following the manual. Genomic DNA (gDNA) was isolated with the traditional phenol-chloroform method. The gDNA samples were treated with RNase A whereas the RNA samples were treated with DNase I, both followed by extraction with phenol and chloroform to remove the enzyme. The DNA or RNA samples were then precipitated and washed with ethanol at a final concentration of 70%.

An aliquot of RNA from each cell line was reverse-transcribed to cDNA with random hexamers and M-MLV Reverse Transcriptase (Promega, Cat. M1705; www.promega.com), following the manual. Forty cycles of PCR were performed to ensure that the reactions entered into the plateau of the amplification of the authentic cDNA and that possible PGs were detectable. PCR products were separated in 1% agarose gel, visualized with ethidium bromide staining, and photographed with Kodak Digital Campture DC290 Camera under a UV light.

## Results

### Identification of PGs of the ACTB, Actb, GAPDH, Gapdh, HPRT1 and Hprt

**Figure 1:**
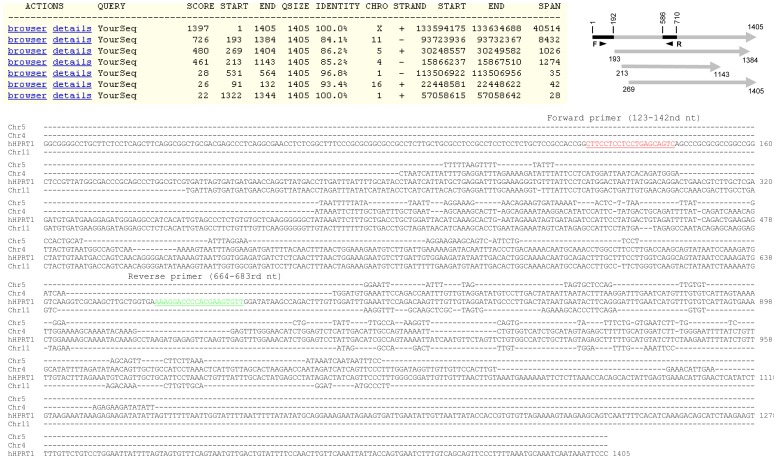
Identification of HPRT1 PGs for primer design. **Top panel**: Blat search using HPRT1 mRNA sequence (1405-bp long after deletion of the poly-A tail) as the bait pulls out three putative PGs, besides the authentic HPRT1 genomic sequence that spans 40514 nt on the plus strand of X chromosome. The three putative PGs match the 193–1383rd, the 269–1413th, and the 213–1143rd nt regions of the HPRT1 mRNA as illustrated. There are three additional very short fragments, spanning only 28, 35 and 35 bp, respectively, that also match parts of HPRT1 mRNA but are not considered as PGs. **Bottom panel**: We pulled out the sequence of each PG (by clicking “details”) and assembled those homologous parts to construct the “cDNA” of the PGs on chromosomes 11 and 4. Alignment of the three sequences with the HPRT1 mRNA reveals that the HPRT1 mRNA has some unique regions. The forward and reverse primers (table 2) we designed are underlined.

**Figure 2:**
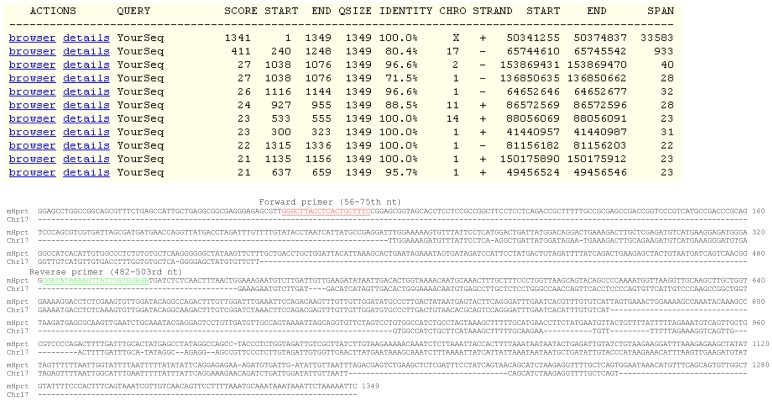
Identification of Hprt PG for primer design. **Top panel**: Blat search using Hprt mRNA sequence as the bait pulls out only one putative PG, besides the authentic Hprt genomic sequence that spans 33583 bp in the plus strand of the mouse X chromosome. This PG matches the 240–1248th nt of the Hprt mRNA and spans 933 nt on the mouse chromosome 17. **Bottom panel**: We pulled out the PG sequence and assembled the parts that are homologous to the Hprt mRNA to construct a cDNA. Alignment of the assembled cDNA with the Hprt mRNA reveals that the Hprt mRNA has several unique regions. The forward and reverse primers (table 2) we designed in some unique regions are underlined.

The mRNA (actually shown as DNA) sequences of the ACTB, Actb, GAPDH, Gapdh, HPRT1 and Hprt, with their gene identity and mRNA access numbers, are shown in figure S1. The GAPDH has an mRNA variant (NM_001256799.1) that is transcribed from an alternative initiation site and thus differs from the wild type mRNA (NM_002046.4) at the 5′-part (Fig. S1). Like many RNA transcripts [19], Actb and Hprt mRNAs contain only poly-A signal but lack a long poly-A tail (Fig. S1), which is a reason for us to perform RT with random hexamers. We used these mRNA sequences, after deleting the poly-A tail from those having it, as a bait to fish out their PGs from the corresponding (human or mouse) genome in the UCSC Genome Browser Database (http://genome.ucsc.edu/) by performing Blat search [31]. The UCSC Genome Browser scores similarity according to not only sequence identity but also sequence length, gap, etc, with a higher score indicating a generally better similarity. The results identified 64, 69, 67, 197, 3 and 1 PGs for the ACTB, Actb, GAPDH, Gapdh, HPRT1 and Hprt, respectively (table 1), which score over 200 and have over 80% identity to the bait. Those genomic sequences that score less than 200 are not counted in, although they still have over 83% identity to the bait and span several hundred nucleotides (nt) on the corresponding chromosome. The details of these PGs, such as their chromosomal locations, sizes, starting and ending nt, homologues, etc, are shown in figures 1 and 2 as well as figures S2, S3, S4 and S5. Most of these PGs are processed, i.e. intronless, as they are similar in size to the bait. Use of other tools such as Blast of the NCBI database (http://blast.ncbi.nlm.nih.gov/Blast.cgi), search from other sources such as the PG database (http://www.pseudogene.org/), or imposition of different criteria for the cutoff may result in different numbers of PGs. For instance, another study identified only 56 PGs of the GAPDH and 166 PGs of the Gapdh [32]. However, the conclusion remains the same that there are many PGs of these genes in the human and mouse genomes.

**Table 1:** Number of putative pseudogenes.

	Human			Mouse		
Gene	ACTB	GAPDH	HPRT1	Actb	Gapdh	Hprt
Number	64	67	3	69	197	1

Note: Only those putative pseudogenes that score over 200 are counted, with details are presented in S-Fig. 2, 3, 4, and 5.

### HPRT1 or Hprt primer design starting from discrimination against PGs

The result of Blat search shows that the HPRT1 mRNA, which is a 1405-bp sequence after its poly-A tail is deleted, spans over 40514 nt in the plus strand of the X chromosome (Fig. 1, top panel). The 93723936–93732367th nt region of the minus strand of chromosome 11 has an 88.3% identity to the 193–1384th nt region of the HPRT1 mRNA. This putative PG spans 8432 nt and is thus unprocessed, i.e. containing intron(s). Another putative PG at chromosome 5 has an 86.2% identity to the 269–1404th nt region of the HPRT1 mRNA. The third putative PG is at chromosome 4 and is homologous to the 213–1143rd nt region of the HPRT1 mRNA; it spans 1274 nt, longer than 930 bp (213–1143rd), and thus may be unprocessed as well. There are three other genomic fragments that are highly homologous to parts of the HPRT1 mRNA but are too small (spanning only 28, 35 and 42 nt, respectively) to be considered as PGs (Fig. 1, top panel).

During Blat search, we clicked the “details” of each PG shown in figure 1 to display the whole sequence. For the unprocessed PGs on chromosomes 11 and 4, we assembled together the parts that are homologous to the HPRT1 mRNA to construct the putative “cDNA”. Alignment of the HPRT1 mRNA with the original sequence or the assembled sequences of the three PGs revealed that the 1–192nd nt and the 586–710th nt regions of the HPRT1 mRNA are lacking in the three PGs (Fig. 1, bottom panel). We designed a forward primer at the 123–142nd nt and a reverse primer at the 664–683rd nt regions (table 2). The HPRT1 mRNA also has other unique regions that may be used for primer design as well (Fig. 1, bottom panel).

**Table 2:** Primer information.

Primer Name	Sequence	Size	Location	Fragment	Region
hACTB-F1452	5′-TTAATAGTCATTCCAAATATGA-3′	22-mers	exon 6	246 bp	1452–1473rd nt
hACTB-R1697	5′-GGGACAAAAAAGGGGGAAGG-3′	20-mers	exon 6		1678–1697th nt
hGAPDH-F6	5′-GAGCCCGCAGCCTCCCGCTT-3′	20-mers	exon 1	700 bp	6–25th nt
hGAPDH-R705	5′-CCCGCGGCCATCACGCCACAG-3′	21-mers	exon 8		685–705th nt
mActb-F1471	5′-GACTTTGTACATTGTTTTG-3′	19-mers	exon 6	382 bp	1471–1489th nt
mActb-R1870	5′-TGCACTTTTATTGGTCTCA-3′	19-mers	exon 6		1870–1852nd nt
hHPRT1-F123	5′-CTTCCTCCTCCTGAGCAGTC-3′	20-mers	exon 1	561 bp	123–142nd nt
hHPRT1-R683	5′-AACACTTCGTGGGGTCCTTT-3′	20-mers	exon 7		664–683rd nt
mHprt1–F56	5′-GGGCTTACCTCACTGCTTTC-3′	20-mers	exon 1	448 bp	56–75th nt
mHprt1–R503	5′-TCTCCACCAATAACTTTTATGTCC-3′	24-mers	exon 4		482–503rd nt

Note: The “h” or “m” in front of each primer's name indicates the human or mouse origin. “F” or “R” indicates a forward or reverse primer. The number after “F” indicates the position of the first nucleotide (nt) of that primer in the mRNA sequence, whereas the number after “R” indicates the position of the last nt of that primer in the mRNA sequence. Thus, the “R” number minuses the “F” number and then pluses one is the size of the DNA fragment amplified by PCR.

Use of the Hprt mRNA sequence as a bait to fish in the mouse genome identified one putative PG that locates at chromosome 17 and is homologous to the 240–1248th nt region of the Hprt mRNA (Fig. 2, top panel). We displayed the sequence of this PG by clicking “details” during Blat search and assembled the parts that are homologous to the Hprt mRNA to construct the “cDNA”. Alignment of this “cDNA” with the Hprt mRNA shows that the 1–240th nt, the 270–535th nt, and several other regions of the Hprt mRNA are unique to the Hprt (Fig. 2, bottom panel). We designed a forward primer at the 56–75th nt and a reverse primer at the 482–503rd nt regions (table 2).

### Design of ACTB, Actb and GAPDH primers that discriminate against PGs

**Figure 3:**
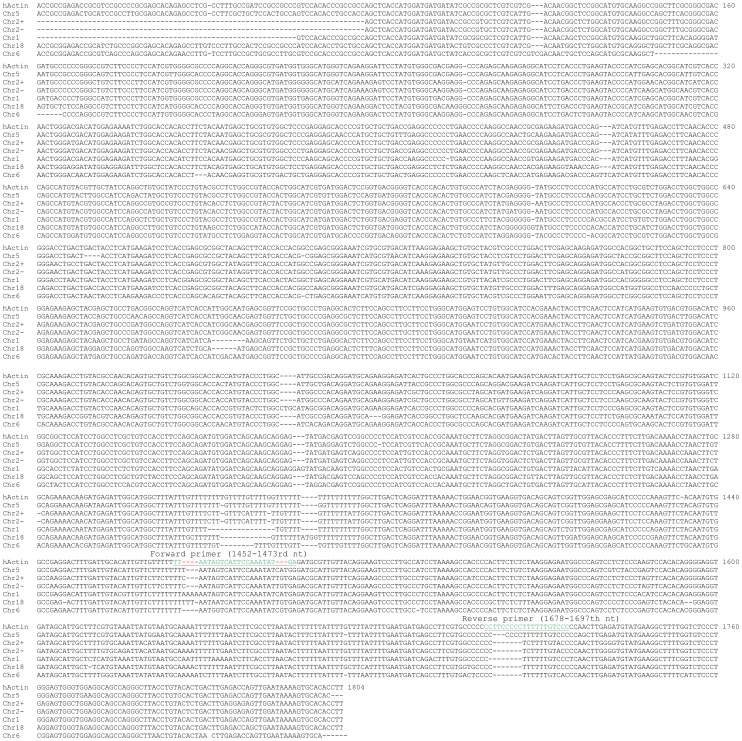
Alignment of the ACTB mRNA with six PGs that are the most homologous to the ACTB mRNA shows that the ACTB mRNA (after deletion of the poly-A tail) has no unique region that is long enough to be a primer. The 1452–1473rd nt and the 1678–1697th nt regions of the ACTB mRNA (underlined) encompass the most mismatches compared with other regions, and thus are selected as the forward and reverse primers, respectively.

By a quick glance at the figure S2, one could immediately realize that most PGs of the ACTB are similar in length to the ACTB mRNA and thus are intronless. The ACTB mRNA lacks a unique region, making it difficult to design primers that would not mis-prime the PGs. We thus pulled out the sequences of six best-scored PGs (in the red box in Fig. S2) and aligned them with the bait sequence. The results confirm that the ACTB mRNA does not contain a unique part that is long enough for a primer (Fig. 3). The best regions we could find for forward and reverse primers that might have some discrimination against the six PGs are the 1452–1473rd nt and the 1678–1697th nt regions of the ACTB mRNA, respectively (Fig. 3 and table 2). However, since there are a total of 64 putative PGs and only six of them were aligned, it remains possible that these primers may match better some of the other PGs than the six aligned.

**Figure 4:**
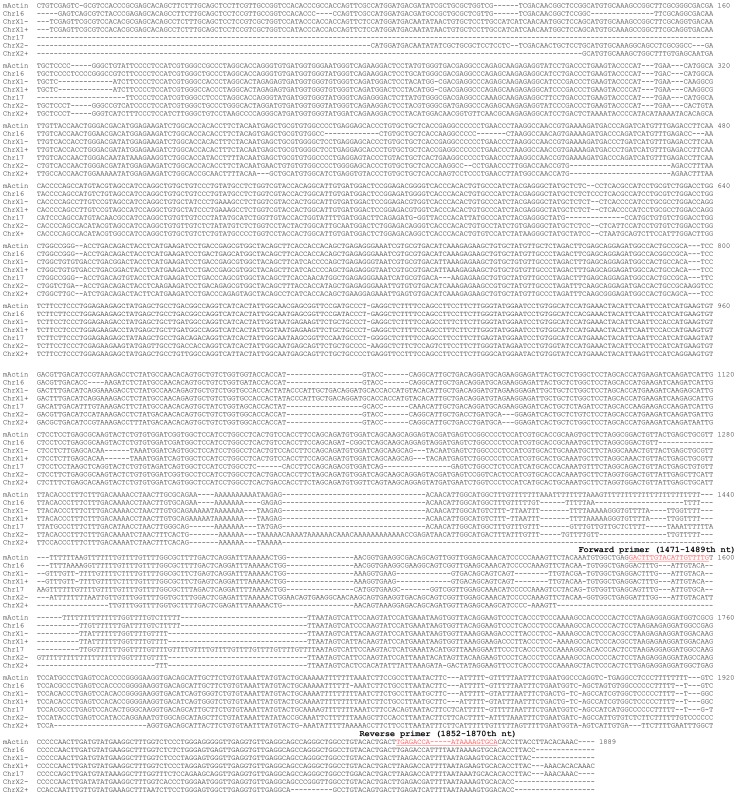
Alignment of the Actb mRNA with the six PGs that are the most homologous to the Actb mRNA shows that the Actb mRNA has no unique region that is long enough to be a primer. The 1471–1489th nt and 1852–1870th nt regions of the Actb mRNA (underlined) have the most mismatches compared with other regions and thus are used as the forward and reverse primers, respectively.

Similar to its human counterpart, the mouse Actb mRNA does not have any unique sequence either, relative to its PGs (Fig. S3). We pulled out the sequences of six best-scored PGs (in the red box in Fig. S3) and aligned them with the Actb mRNA. The results confirm that the Actb mRNA has no unique part that is long enough to be a primer (Fig. 4). Nevertheless, we selected the 1471–1489th nt and the 1852–1869th nt regions of the Actb mRNA as forward and reverse primers, respectively (table 2), which might better discriminate against the six PGs than the other parts of the Actb mRNA, although it remains possible that these primers may match better some of the other PGs than the six aligned.

**Figure 5:**
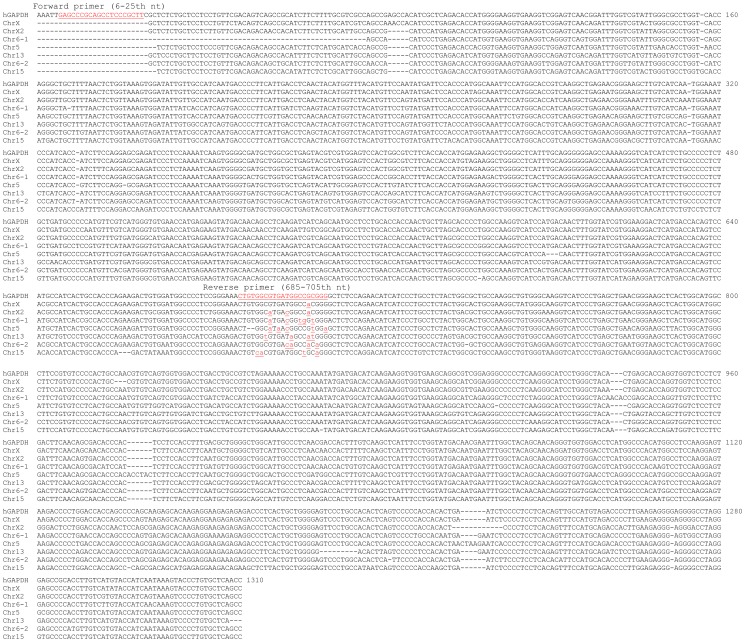
Alignment of the human GAPDH mRNA with seven PGs that are the best homologous to the wild type GAPDH mRNA shows that the first 26 nt of the mRNA is the only unique region while the 685–705th nt region of the GAPDH has the most mismatches (shown as underlined lowercase letters in PGs). We select the 6–25th and the 685–705th regions as the forward and reverse primers (underlined), respectively.

By a quick glance at figure S4, one could immediately find that the first 26 nt of the wild type GAPDH mRNA is a unique region. We pulled out the sequences of seven best-scored PGs (in the red box in Fig. S4) and aligned them with the GAPDH mRNA. The results not only confirm the uniqueness of the 1–26th nt but also show that the 685–705th nt region has the most mismatches to most PGs, except the first X-linked PG that only has one nt mismatched to the GAPDH (Fig. 5). We used these two regions as the forward and reverse primers, respectively (table 2), in part because this pair of primer will not amplify the variant 2 of GAPDH (NM_001256799.1), transcriptional feature of which is unknown.

### Impossibility of designing Gapdh specific primers

**Figure 6:**
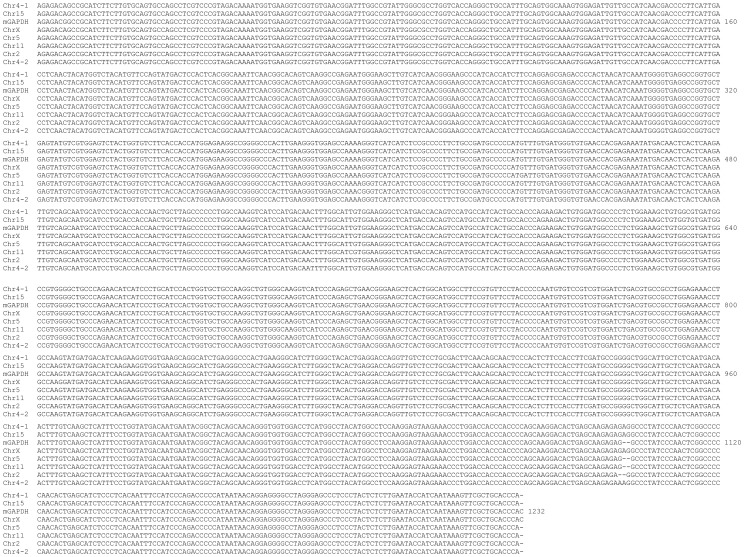
Alignment of the mouse Gapdh mRNA with seven PGs that are the best homologous to the Gapdh mRNA shows that these PGs are almost identical to the Gapdh with only several mismatches. No region of the Gapdh can be used as a primer that can significantly discriminate against any of the PGs.

A quick glance at figure S5 immediately leads us to the fact that there are so many processed PGs of the mouse Gapdh which are 100% or almost 100% identical to the Gapdh mRNA. Indeed, alignment of the Gapdh mRNA with seven best-scored PGs (in the red box in Fig. S5) showed several-nt mismatches only, making it impossible to design any primer that can discriminate against the PGs (Fig. 6).

### Verification of the ACTB, GAPDH and HPRT1 primers

**Figure 7:**
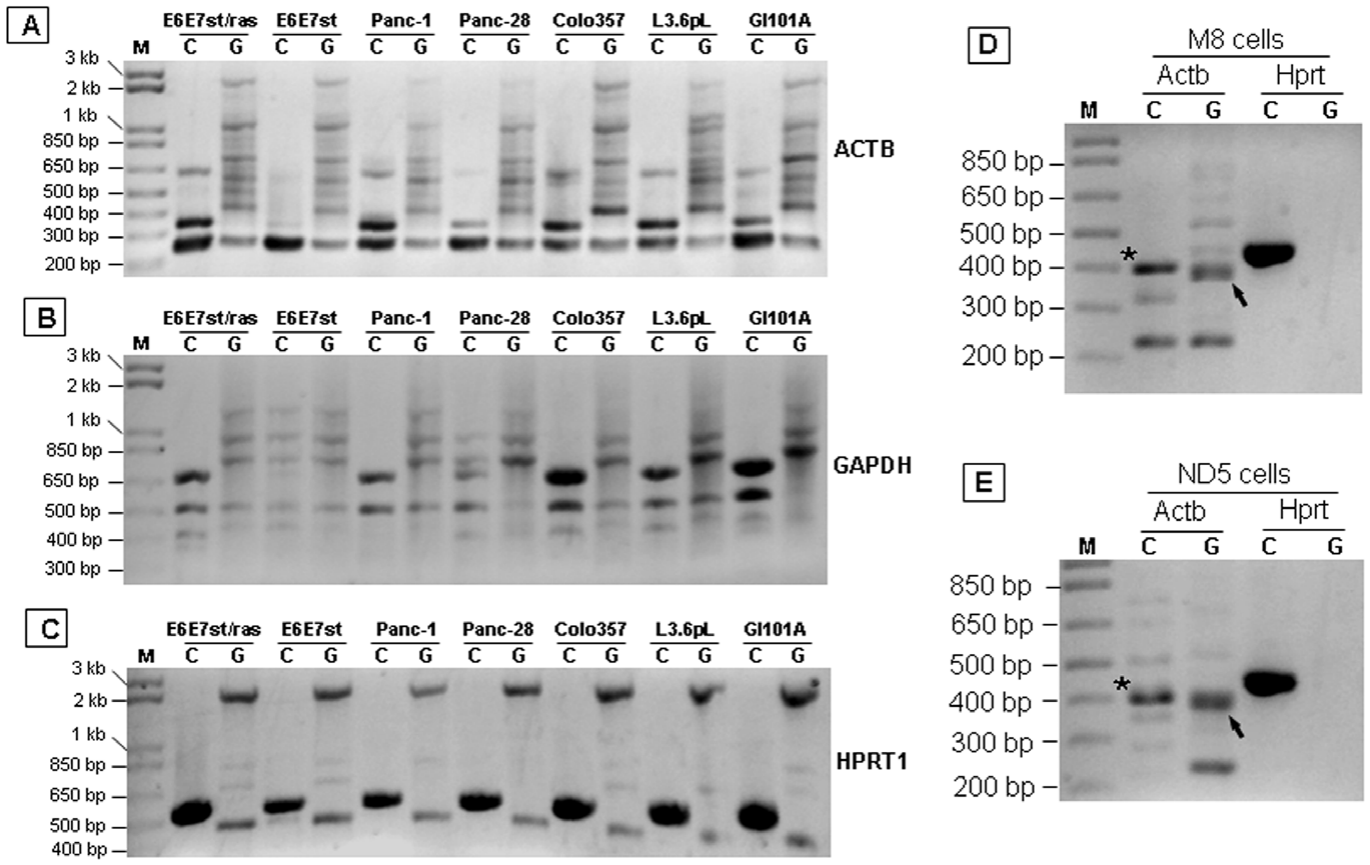
Determination of the primer specificity for PCR both vertically and horizontally. **A–C**: gDNA (G) and cDNA (C) samples from a panel of human cell lines (described in materials and methods) were amplified by PCR with conditions of initial denature at 95°C for 5 min and 40 cycles of melting at 95°C for 30 sec, primer-annealing at 58°C for 30 sec, and elongating at 72°C for 30 sec. The reaction was determined at 72°C for 10 min. M is molecular weight marker. **D** and **E**: gDNA (G) and cDNA (C) samples from M8 and ND5 mouse cell lines were amplified by PCR under the conditions described above. Stars indicate the authentic Actb cDNA band whereas arrows indicate its counterpart from gDNA samples.

PCR results showed that the authentic ACTB band of 246 bp (table 2) in agarose gel was amplified easily, as expected, in the cDNA sample from a panel of human cell lines (Fig. 7A). Although both forward and reverse primers locate at the same exon (exon 6) and thus should also amplify the authentic gene, the same band was detected only weakly (not stronger than nonspecific bands) from the gDNA sample of the same cell lines (Fig. 7A). Thus, this pair of primers meets our purpose to discriminate against gDNA, both the authentic gene and the PGs, as discussed later. However, an additional band that was about 100-bp larger (∼350 bp) than the 246-bp ACTB cDNA and another one of about 650 bp were also amplified from the cDNA (but not the gDNA) samples, suggesting that our primers also mis-prime cDNA of two unknown genes, expression of which, as expected, varied among different cell lines, as manifested by different ratios to the 246-bp ACTB band that serves as the internal reference in the same cell line (Fig. 7A). This pair of primers also detected many nonspecific bands from gDNA samples that differed in size from the 246-bp band.

The authentic band of the wild type GAPDH (700-bp) was detected only in the cDNA, but not the gDNA, samples, although the primers also detected cDNA of two unknown genes at smaller (∼500 and 400 bp, respectively) sizes and detected several gDNA fragments of different sizes (Fig. 7B). Our HPRT1 primers amplified only the anticipated band (at 561-bp) from cDNA samples, although the primers also amplified several bands of different sizes from gDNA samples (Fig. 7C).

We had obtained similar results from cDNA and gDNA samples of many other human cell lines and tissues by using the same ACTB, GAPDH and HPRT1 primers (data not shown). Based on our experience, the PCR conditions for these primers are recommended as initial denature at 95°C for 5 min, followed by each cycle of melting at 95°C for 30 sec, primer-annealing at 58°C for 30 sec, and elongating at 72°C for 30 sec. To maintain the reaction at the linear portion, the number of cycles should be significantly decreased to less than 30 cycles, unless a very small amount of cDNA template is used. The reaction should be terminated at 72°C for 10 min.

### Verification of the Actb and Hprt primers

Our Hprt primers (table 2) amplified only the anticipated band from the cDNA sample, without mis-priming gDNA, from the M8 and ND5 mouse cell lines, thus confirming the specificity of the primers (Fig. 7D and 7E). In contrast, our Actb primers (table 2) produced not only the authentic Actb cDNA band of 382-bp (star in Fig. 7D and 7E) but also several other bands that were less abundant and differed between the cDNA samples from M8 and ND5 cells, indicating that the primers also mis-prime cDNA of other unknown genes expression of which differs between cell lines. gDNA samples also produced a band that was similar in size to the Actb cDNA but was very fuzzy, likely because it was a mixture of different bands, including some that were actually slightly smaller than the Actb cDNA (arrow in Fig. 7D and 7E). Likely, this or these fuzzy bands are derived also from some PGs, besides the authentic Actb gene that should be amplified as both primers locate at the same exon (exon 6). The Actb primers also amplified other bands from gDNA samples that differed between the two cell lines as well (Fig. 7D and 7E). We had also studied many other mouse cell lines and tissues and obtained very similar results (data not shown).

## Discussion

Our bioinformatic analyses show that the ACTB (Actb) and GAPDH (Gapdh) genes have 64–197 putative PGs (table 1) that score over 200 and have over 80% identity to the corresponding parental mRNA, based on the UCSC Genome Browser [31]. There are some more genomic fragments scored lower than 200 and thus not accounted in as PGs, but they span over several hundred nt, have over 83% identity to the authentic mRNA, and may still be mis-primed. If, like its 1% that has been studied [20], the human genome has 93% of its bases being transcribed, we may have to accept the new concept that most of the genome is transcribed at least in some cell types or at some times. Because each of these PGs resides at a different chromosomal site from the authentic gene, if any of them is expressed, it is controlled by different transcription-regulatory elements and thus is actually a different gene. Therefore, before the ACTB (Actb) or GAPDH (Gapdh) can be used as a reference in RT-PCR, it needs to be confirmed that none of their 64–197 putative PGs is transcribed in the particular cell (tissue) or situation of interest. To determine whether many processed PGs are expressed or not, the only strategy we can think of is to clone the RT-PCR products from each interested cell line or tissue into a vector, followed by sequencing a large number of plasmid clones to ensure that none of the clones has a PG sequence. If some PGs are found to be expressed, how their transcription is regulated needs to be determined, which needs to use another gene as the reference, so as to determine whether they meet the criteria of a reference gene, including refractoriness to the to-be-studied situation. Without saying, it is practically impossible to perform such a tedious and cumbersome sideshow to determine the expression status of so many PGs and to determine their transcriptional features, especially when a study involves multiple cell lines (tissues) or multiple experimental situations. It is much simpler to forgo these genes and elect someone else, such as the HPRT1 or Hprt.

In eukaryotic genomes, 2.7–97.7% of the genes are intronless [33], such as about 50% of the G protein coupled receptor genes in the human [34], while many other genes have processed PGs. RT-PCR amplification of the RNA transcripts from these two classes of genes requires a perfect DNase digestion to remove not only genomic DNA residuals but also mitochondrial DNA from the RNA sample, since some chromosomal genes are highly homologous to their ancestors in mitochondria [35], [36]. According to our experience, complete DNase digestion that leaves no traceable DNA residual in the RNA sample is actually not so easy, because PCR is supersensitive. PG may cause artifact without being transcribed, which can be exemplified by the Gapdh: any DNA residual that survives the DNase treatment would, theoretically, amplify 198 times of the template (1 parental gene plus 197 PGs), which is then increased exponentially in PCR, although usually gDNA is more difficult to be amplified as discussed later. This is another reason for forgoing those genes with many processed PGs as a reference, even if the PGs are not transcribed or specific primers can be designed, such as the GAPDH. We usually assign forward and reverse primers to two different exons with one or more introns in between, because in this way the gDNA is either too lengthy to be amplified by PCR or is amplified as a fragment larger than the cDNA. Some published studies omitted DNase digestion of RNA samples in part because the primers were designed in this way, but it may not be ideal since the primers cannot distinguish the cDNA from intronless genes and processed PGs because their sizes are identical or similar to that of the cDNA.

With the above explained, we propose a standard operation procedure (SOP) for discussion in which design of primers for RT-PCR start from exclusion of mis-priming PGs, since so many genes have them [21]. The simplest way is to use the to-be-studied mRNA sequence, after removing its poly-A tail if there is one, as a bait to fish out PGs from the corresponding genome. Primers should be assigned to the regions unique to the mRNA, such as the first 26 nt of the wild type GAPDH (NM_002046.4). If there is no unique region, a second step should be taken to align the mRNA with the PG sequence, so as to identify regions that encompass the most mismatches, such as the region used as our GAPDH reverse primer. Once such a region is selected, the whole mRNA sequence, but not just the selected region, should be used to run the routine primer designing software to further analyze whether a primer can be identified within the selected region. It merits mention that one type of quantitative PCR technique uses a gene-specific oligo (with modified backbone) probe, besides the two primers, to increase the specificity. This strategy should also enhance the preference to the authentic cDNA if, like the two primers, the probe is also assigned to a region containing mismatches. However, it remains possible that like the two primers, the probe also mis-anneals with some PGs, due to the high sequence similarity. It also merits mention that because mouse genome varies hugely among different strains [37], the real risk of mis-priming may be higher in the cells or tissues from some strains of mice.

Specificity is an important criterion of primer design for PCR, which hitherto is concerned only vertically: appearance of additional band(s) above or below the expected one(s) in the same lane of the agarose gel is indicative of non-specificity of the primer pair, as seen in the PCR results from our ACTB and Actb primers (Fig. 7A, 7D and 7E). We now propose to consider the specificity also horizontally as part of the SOP: gDNA sample should be included in PCR as template and in the ensuing gel electrophoresis, so as to determine whether there are processed PGs amplified. This is needed if it is preferred not to determine whether a PG is transcribed in the to-be-studied cell type or situation. If the expected band appears also in the gDNA samples, the primers need to be redesigned or a complete DNase digestion of RNA sample needs to be ensured. As an example of the horizontal criteria, the slight difference in the expected band of Actb between cDNA and gDNA samples is discerned when they were loaded into the agarose gel in a side-by-side manner, i.e. the band from gDNA samples is fuzzy and seems to be mixed bands (Fig. 7D and 7E), suggesting that the primers may not be specific horizontally, although they were selected already under the best consideration of PGs. A caveat needs to be given that it is generally more difficult to perform PCR with gDNA as template, in part because long chromatin DNAs are highly wound and difficult to be denatured by heating and annealed by primers. This may be one of the reasons why results from gDNA samples may differ among different cell lines for our ACTB and GAPDH primers and why our ACTB primers, both of which locate at the same exon (exon 6), seem to amplify cDNA samples more easily than gDNA samples (Fig. 7A). PCR results of the ACTB (Actb) and GAPDH (Gapdh) shown in the literature, including those from us previously, may not be specific if evaluated horizontally. Indeed, we randomly performed bioinformatic analyses of quite a few PCR primers of these genes reported in the three most prestigious journals, i.e. Nature, Science and Cell; without exception, all those primers match well and amplify the corresponding PGs by insilico PCR. Whether or not the previously published RT-PCR results that involve these genes (especially the Gapdh) as the reference need to be reevaluated or reinterpreted is an uncomfortable but unavoidable question, and should be left to the corresponding authors to decide accordingly.

An additional reason to abandon ACTB is that the DNA fragment amplified by our primer pair, which is the only one we can find in consideration of the specificity horizontally, is only 246-bp long (table 2). This size may be suitable for real-time RT-PCR but is too short to sensitively reflect a difference in the copy numbers of the ACTB mRNA, if the RT-PCR products are evaluated by visualization in an agarose gel, because it requires more copies of a small DNA fragment to reach a visible amount of nucleotides in a gel. For instance, one more copy of a 246-bp double-stranded DNA fragment only adds 492 nucleotides in the gel, which is not as visible as one more copy of a 1-kb DNA fragment that adds 2,000 nucleotides. For this technical reason, we usually set, if possible, primer pairs to amplify DNA fragments of 400–700 bp and to minimize the difference in the fragment sizes between the reference gene and the interested gene.

In summary, in this study we provide bioinformatic data showing that the genes encoding β-actin and glyceraldehyde-3-phosphate dehydrogenase have many PGs in the human and mouse genomes. These PGs may affect the fidelity of ACTB (Actb) or GAPDH (Gapdh) as a reference in RT-PCR by their genomic DNA or, if some of them are expressed, by their RNA transcript, because a large copy number in the genome may amplify the artifact derived from genomic DNA residual in the RNA sample during PCR whereas their RNA transcript may contribute to the yield of RT-PCR. We suggest to peers to forgo these genes, especially the Gapdh, as a reference in RT-PCR or, if there is no suitable surrogate, to use with extra caution our primers and PCR conditions provided herein that may better avoid mis-priming PGs, relative to most primers described in the literature. We also propose an SOP in which design of primers for RT-PCR starts from avoiding mis-priming PGs and all primers need be tested with not only cDNA but also gDNA to ensure their specificity both vertically and horizontally.

## Supporting Information

Figure S1 ACTB, Actb, GADPH, Gadph, HPRT1 and Hprt mRNA sequences pulled out from the NCBI database that presents all mRNA as DNA sequence, i.e. with thymine replacing uracil.
(DOC)

Figure S2 Putative PGs of the ACTB identified by Blat search using the ACTB mRNA sequence (after deletion of the poly-A tail). The top sequence that has 100% identity to the bait is the authentic ACTB gene on chromosome 7. The six genomic DNA fragments that have the highest scores to the bait were used in the alignment with the bait sequence shown in figure 3.
(DOC)

Figure S3 Putative PGs of the Actb identified by Blat search using the Actb mRNA sequence. The top sequence that has 100% identity to the bait is the authentic Actb gene on mouse chromosome 5. The six genomic DNA fragments in the red box that have the highest scores to the bait were used in the alignment with the bait sequence shown in figure 4.
(DOC)

Figure S4 Putative PGs of the GAPDH identified by Blat search using the GAPDH mRNA sequence (after deletion of the poly-A tail). The top sequence that has 100% identity to the bait is the authentic GAPDH gene on human chromosome 12. The seven genomic DNA fragments in the red box that have the highest scores to the bait were used in the alignment with the bait sequence shown in figure 5.
(DOC)

Figure S5 Putative PGs of the Gapdh identified by Blat search using the Gapdh mRNA sequence (after deletion of the poly-A tail). The top sequence that has 100% identity to the bait is the authentic Gapdh gene on the mouse chromosome 7. The seven genomic DNA fragments in the red box that have the highest scores to the bait were used in the alignment with the bait sequence shown in figure 6.
(DOC)
