# Supplementary material for: Pseudogenes as Weaknesses of ACTB (Actb) and GAPDH (Gapdh) Used as Reference Genes in Reverse Transcription and Polymerase Chain Reactions
Source: PLoS One. 2012 Aug 22;7(8):e41659. doi: 10.1371/journal.pone.0041659 (PMC3425558; doi:10.1371/journal.pone.0041659)
Supplement: Figure S1 — ACTB, Actb, GADPH, Gadph, HPRT1 and Hprt mRNA sequences pulled out from the NCBI database that presents all mRNA as DNA sequence, i.e. with thymine replacing uracil. (DOC) [file pone.0041659.s001.doc]

**Human genes**

Human ACTB mRNA:

>gi|168480144|ref|NM_001101.3| Homo sapiens actin, beta (ACTB), mRNA:

ACCGCCGAGACCGCGTCCGCCCCGCGAGCACAGAGCCTCGCCTTTGCCGATCCGCCGCCCGTCCACACCCGCCGCCAGCTCACCATGGATGATGATATCGCCGCGCTCGTCGTCGACAACGGCTCCGGCATGTGCAAGGCCGGCTTCGCGGGCGACGATGCCCCCCGGGCCGTCTTCCCCTCCATCGTGGGGCGCCCCAGGCACCAGGGCGTGATGGTGGGCATGGGTCAGAAGGATTCCTATGTGGGCGACGAGGCCCAGAGCAAGAGAGGCATCCTCACCCTGAAGTACCCCATCGAGCACGGCATCGTCACCAACTGGGACGACATGGAGAAAATCTGGCACCACACCTTCTACAATGAGCTGCGTGTGGCTCCCGAGGAGCACCCCGTGCTGCTGACCGAGGCCCCCCTGAACCCCAAGGCCAACCGCGAGAAGATGACCCAGATCATGTTTGAGACCTTCAACACCCCAGCCATGTACGTTGCTATCCAGGCTGTGCTATCCCTGTACGCCTCTGGCCGTACCACTGGCATCGTGATGGACTCCGGTGACGGGGTCACCCACACTGTGCCCATCTACGAGGGGTATGCCCTCCCCCATGCCATCCTGCGTCTGGACCTGGCTGGCCGGGACCTGACTGACTACCTCATGAAGATCCTCACCGAGCGCGGCTACAGCTTCACCACCACGGCCGAGCGGGAAATCGTGCGTGACATTAAGGAGAAGCTGTGCTACGTCGCCCTGGACTTCGAGCAAGAGATGGCCACGGCTGCTTCCAGCTCCTCCCTGGAGAAGAGCTACGAGCTGCCTGACGGCCAGGTCATCACCATTGGCAATGAGCGGTTCCGCTGCCCTGAGGCACTCTTCCAGCCTTCCTTCCTGGGCATGGAGTCCTGTGGCATCCACGAAACTACCTTCAACTCCATCATGAAGTGTGACGTGGACATCCGCAAAGACCTGTACGCCAACACAGTGCTGTCTGGCGGCACCACCATGTACCCTGGCATTGCCGACAGGATGCAGAAGGAGATCACTGCCCTGGCACCCAGCACAATGAAGATCAAGATCATTGCTCCTCCTGAGCGCAAGTACTCCGTGTGGATCGGCGGCTCCATCCTGGCCTCGCTGTCCACCTTCCAGCAGATGTGGATCAGCAAGCAGGAGTATGACGAGTCCGGCCCCTCCATCGTCCACCGCAAATGCTTCTAGGCGGACTATGACTTAGTTGCGTTACACCCTTTCTTGACAAAACCTAACTTGCGCAGAAAACAAGATGAGATTGGCATGGCTTTATTTGTTTTTTTTGTTTTGTTTTGGTTTTTTTTTTTTTTTTGGCTTGACTCAGGATTTAAAAACTGGAACGGTGAAGGTGACAGCAGTCGGTTGGAGCGAGCATCCCCCAAAGTTCACAATGTGGCCGAGGACTTTGATTGCACATTGTTGTTTTTTTAATAGTCATTCCAAATATGAGATGCGTTGTTACAGGAAGTCCCTTGCCATCCTAAAAGCCACCCCACTTCTCTCTAAGGAGAATGGCCCAGTCCTCTCCCAAGTCCACACAGGGGAGGTGATAGCATTGCTTTCGTGTAAATTATGTAATGCAAAATTTTTTTAATCTTCGCCTTAATACTTTTTTATTTTGTTTTATTTTGAATGATGAGCCTTCGTGCCCCCCCTTCCCCCTTTTTTGTCCCCCAACTTGAGATGTATGAAGGCTTTTGGTCTCCCTGGGAGTGGGTGGAGGCAGCCAGGGCTTACCTGTACACTGACTTGAGACCAGTTGAATAAAAGTGCACACCTTAAAAATGAAAAAAAAAAAAAAAAAAAAAAAAAAAAAAAAAAAAAAAAA

Human GAPDH mRNA:

>gi|83641890|ref|NM_002046.4| Homo sapiens glyceraldehyde-3-phosphate dehydrogenase (GAPDH), mRNA:

GGCTGGGACTGGCTGAGCCTGGCGGGAGGCGGGGTCCGAGTCACCGCCTGCCGCCGCGCCCCCGGTTTCTATAAATTGAGCCCGCAGCCTCCCGCTTCGCTCTCTGCTCCTCCTGTTCGACAGTCAGCCGCATCTTCTTTTGCGTCGCCAGCCGAGCCACATCGCTCAGACACCATGGGGAAGGTGAAGGTCGGAGTCAACGGATTTGGTCGTATTGGGCGCCTGGTCACCAGGGCTGCTTTTAACTCTGGTAAAGTGGATATTGTTGCCATCAATGACCCCTTCATTGACCTCAACTACATGGTTTACATGTTCCAATATGATTCCACCCATGGCAAATTCCATGGCACCGTCAAGGCTGAGAACGGGAAGCTTGTCATCAATGGAAATCCCATCACCATCTTCCAGGAGCGAGATCCCTCCAAAATCAAGTGGGGCGATGCTGGCGCTGAGTACGTCGTGGAGTCCACTGGCGTCTTCACCACCATGGAGAAGGCTGGGGCTCATTTGCAGGGGGGAGCCAAAAGGGTCATCATCTCTGCCCCCTCTGCTGATGCCCCCATGTTCGTCATGGGTGTGAACCATGAGAAGTATGACAACAGCCTCAAGATCATCAGCAATGCCTCCTGCACCACCAACTGCTTAGCACCCCTGGCCAAGGTCATCCATGACAACTTTGGTATCGTGGAAGGACTCATGACCACAGTCCATGCCATCACTGCCACCCAGAAGACTGTGGATGGCCCCTCCGGGAAACTGTGGCGTGATGGCCGCGGGGCTCTCCAGAACATCATCCCTGCCTCTACTGGCGCTGCCAAGGCTGTGGGCAAGGTCATCCCTGAGCTGAACGGGAAGCTCACTGGCATGGCCTTCCGTGTCCCCACTGCCAACGTGTCAGTGGTGGACCTGACCTGCCGTCTAGAAAAACCTGCCAAATATGATGACATCAAGAAGGTGGTGAAGCAGGCGTCGGAGGGCCCCCTCAAGGGCATCCTGGGCTACACTGAGCACCAGGTGGTCTCCTCTGACTTCAACAGCGACACCCACTCCTCCACCTTTGACGCTGGGGCTGGCATTGCCCTCAACGACCACTTTGTCAAGCTCATTTCCTGGTATGACAACGAATTTGGCTACAGCAACAGGGTGGTGGACCTCATGGCCCACATGGCCTCCAAGGAGTAAGACCCCTGGACCACCAGCCCCAGCAAGAGCACAAGAGGAAGAGAGAGACCCTCACTGCTGGGGAGTCCCTGCCACACTCAGTCCCCCACCACACTGAATCTCCCCTCCTCACAGTTGCCATGTAGACCCCTTGAAGAGGGGAGGGGCCTAGGGAGCCGCACCTTGTCATGTACCATCAATAAAGTACCCTGTGCTCAACCAAAAAAAAAAAAAAAAAAA

Human GAPDH variant 2; NM_001256799.1:

GTGCGCAGCGGGTGCATCCCTGTCCGGATGCTGCGCCTGCGGTAGAGCGGCCGCCATGTTGCAACCGGGAAGGAAATGAATGGGCAGCCGTTAGGAAAGCCTGCCGGTGACTAACCCTGCGCTCCTGCCTCGATGGGTGGAGTCGCGTGTGGCGGGGAAGTCAGGTGGAGCGAGGCTAGCTGGCCCGATTTCTCCTCGGGTGATGCTTTTCCTAGATTATTCTCTGATTTGGTCGTATTGGGCGCCTGGTCACCAGGGCTGCTTTTAACTCTGGTAAAGTGGATATTGTTGCCATCAATGACCCCTTCATTGACCTCAACTACATGGTTTACATGTTCCAATATGATTCCACCCATGGCAAATTCCATGGCACCGTCAAGGCTGAGAACGGGAAGCTTGTCATCAATGGAAATCCCATCACCATCTTCCAGGAGCGAGATCCCTCCAAAATCAAGTGGGGCGATGCTGGCGCTGAGTACGTCGTGGAGTCCACTGGCGTCTTCACCACCATGGAGAAGGCTGGGGCTCATTTGCAGGGGGGAGCCAAAAGGGTCATCATCTCTGCCCCCTCTGCTGATGCCCCCATGTTCGTCATGGGTGTGAACCATGAGAAGTATGACAACAGCCTCAAGATCATCAGCAATGCCTCCTGCACCACCAACTGCTTAGCACCCCTGGCCAAGGTCATCCATGACAACTTTGGTATCGTGGAAGGACTCATGACCACAGTCCATGCCATCACTGCCACCCAGAAGACTGTGGATGGCCCCTCCGGGAAACTGTGGCGTGATGGCCGCGGGGCTCTCCAGAACATCATCCCTGCCTCTACTGGCGCTGCCAAGGCTGTGGGCAAGGTCATCCCTGAGCTGAACGGGAAGCTCACTGGCATGGCCTTCCGTGTCCCCACTGCCAACGTGTCAGTGGTGGACCTGACCTGCCGTCTAGAAAAACCTGCCAAATATGATGACATCAAGAAGGTGGTGAAGCAGGCGTCGGAGGGCCCCCTCAAGGGCATCCTGGGCTACACTGAGCACCAGGTGGTCTCCTCTGACTTCAACAGCGACACCCACTCCTCCACCTTTGACGCTGGGGCTGGCATTGCCCTCAACGACCACTTTGTCAAGCTCATTTCCTGGTATGACAACGAATTTGGCTACAGCAACAGGGTGGTGGACCTCATGGCCCACATGGCCTCCAAGGAGTAAGACCCCTGGACCACCAGCCCCAGCAAGAGCACAAGAGGAAGAGAGAGACCCTCACTGCTGGGGAGTCCCTGCCACACTCAGTCCCCCACCACACTGAATCTCCCCTCCTCACAGTTGCCATGTAGACCCCTTGAAGAGGGGAGGGGCCTAGGGAGCCGCACCTTGTCATGTACCATCAATAAAGTACCCTGTGCTCAACCAAAAAAAAAAAAAAAAAAA

Human HPRT1 mRNA:

>gi|164518913|ref|NM_000194.2| Homo sapiens hypoxanthine phosphoribosyltransferase 1 (HPRT1), mRNA:

GGCGGGGCCTGCTTCTCCTCAGCTTCAGGCGGCTGCGACGAGCCCTCAGGCGAACCTCTCGGCTTTCCCGCGCGGCGCCGCCTCTTGCTGCGCCTCCGCCTCCTCCTCTGCTCCGCCACCGGCTTCCTCCTCCTGAGCAGTCAGCCCGCGCGCCGGCCGGCTCCGTTATGGCGACCCGCAGCCCTGGCGTCGTGATTAGTGATGATGAACCAGGTTATGACCTTGATTTATTTTGCATACCTAATCATTATGCTGAGGATTTGGAAAGGGTGTTTATTCCTCATGGACTAATTATGGACAGGACTGAACGTCTTGCTCGAGATGTGATGAAGGAGATGGGAGGCCATCACATTGTAGCCCTCTGTGTGCTCAAGGGGGGCTATAAATTCTTTGCTGACCTGCTGGATTACATCAAAGCACTGAATAGAAATAGTGATAGATCCATTCCTATGACTGTAGATTTTATCAGACTGAAGAGCTATTGTAATGACCAGTCAACAGGGGACATAAAAGTAATTGGTGGAGATGATCTCTCAACTTTAACTGGAAAGAATGTCTTGATTGTGGAAGATATAATTGACACTGGCAAAACAATGCAGACTTTGCTTTCCTTGGTCAGGCAGTATAATCCAAAGATGGTCAAGGTCGCAAGCTTGCTGGTGAAAAGGACCCCACGAAGTGTTGGATATAAGCCAGACTTTGTTGGATTTGAAATTCCAGACAAGTTTGTTGTAGGATATGCCCTTGACTATAATGAATACTTCAGGGATTTGAATCATGTTTGTGTCATTAGTGAAACTGGAAAAGCAAAATACAAAGCCTAAGATGAGAGTTCAAGTTGAGTTTGGAAACATCTGGAGTCCTATTGACATCGCCAGTAAAATTATCAATGTTCTAGTTCTGTGGCCATCTGCTTAGTAGAGCTTTTTGCATGTATCTTCTAAGAATTTTATCTGTTTTGTACTTTAGAAATGTCAGTTGCTGCATTCCTAAACTGTTTATTTGCACTATGAGCCTATAGACTATCAGTTCCCTTTGGGCGGATTGTTGTTTAACTTGTAAATGAAAAAATTCTCTTAAACCACAGCACTATTGAGTGAAACATTGAACTCATATCTGTAAGAAATAAAGAGAAGATATATTAGTTTTTTAATTGGTATTTTAATTTTTATATATGCAGGAAAGAATAGAAGTGATTGAATATTGTTAATTATACCACCGTGTGTTAGAAAAGTAAGAAGCAGTCAATTTTCACATCAAAGACAGCATCTAAGAAGTTTTGTTCTGTCCTGGAATTATTTTAGTAGTGTTTCAGTAATGTTGACTGTATTTTCCAACTTGTTCAAATTATTACCAGTGAATCTTTGTCAGCAGTTCCCTTTTAAATGCAAATCAATAAATTCCCAAAAATTTAAAAAAAAAAAAAAAAAAAAAA

**Mouse genes**

Mouse Actb mRNA:

>gi|145966868|ref|NM_007393.3| Mus musculus actin, beta (Actb), mRNA:

CTGTCGAGTCGCGTCCACCCGCGAGCACAGCTTCTTTGCAGCTCCTTCGTTGCCGGTCCACACCCGCCACCAGTTCGCCATGGATGACGATATCGCTGCGCTGGTCGTCGACAACGGCTCCGGCATGTGCAAAGCCGGCTTCGCGGGCGACGATGCTCCCCGGGCTGTATTCCCCTCCATCGTGGGCCGCCCTAGGCACCAGGGTGTGATGGTGGGAATGGGTCAGAAGGACTCCTATGTGGGTGACGAGGCCCAGAGCAAGAGAGGTATCCTGACCCTGAAGTACCCCATTGAACATGGCATTGTTACCAACTGGGACGACATGGAGAAGATCTGGCACCACACCTTCTACAATGAGCTGCGTGTGGCCCCTGAGGAGCACCCTGTGCTGCTCACCGAGGCCCCCCTGAACCCTAAGGCCAACCGTGAAAAGATGACCCAGATCATGTTTGAGACCTTCAACACCCCAGCCATGTACGTAGCCATCCAGGCTGTGCTGTCCCTGTATGCCTCTGGTCGTACCACAGGCATTGTGATGGACTCCGGAGACGGGGTCACCCACACTGTGCCCATCTACGAGGGCTATGCTCTCCCTCACGCCATCCTGCGTCTGGACCTGGCTGGCCGGGACCTGACAGACTACCTCATGAAGATCCTGACCGAGCGTGGCTACAGCTTCACCACCACAGCTGAGAGGGAAATCGTGCGTGACATCAAAGAGAAGCTGTGCTATGTTGCTCTAGACTTCGAGCAGGAGATGGCCACTGCCGCATCCTCTTCCTCCCTGGAGAAGAGCTATGAGCTGCCTGACGGCCAGGTCATCACTATTGGCAACGAGCGGTTCCGATGCCCTGAGGCTCTTTTCCAGCCTTCCTTCTTGGGTATGGAATCCTGTGGCATCCATGAAACTACATTCAATTCCATCATGAAGTGTGACGTTGACATCCGTAAAGACCTCTATGCCAACACAGTGCTGTCTGGTGGTACCACCATGTACCCAGGCATTGCTGACAGGATGCAGAAGGAGATTACTGCTCTGGCTCCTAGCACCATGAAGATCAAGATCATTGCTCCTCCTGAGCGCAAGTACTCTGTGTGGATCGGTGGCTCCATCCTGGCCTCACTGTCCACCTTCCAGCAGATGTGGATCAGCAAGCAGGAGTACGATGAGTCCGGCCCCTCCATCGTGCACCGCAAGTGCTTCTAGGCGGACTGTTACTGAGCTGCGTTTTACACCCTTTCTTTGACAAAACCTAACTTGCGCAGAAAAAAAAAAAATAAGAGACAACATTGGCATGGCTTTGTTTTTTTAAATTTTTTTTAAAGTTTTTTTTTTTTTTTTTTTTTTTTTTTTTAAGTTTTTTTGTTTTGTTTTGGCGCTTTTGACTCAGGATTTAAAAACTGGAACGGTGAAGGCGACAGCAGTTGGTTGGAGCAAACATCCCCCAAAGTTCTACAAATGTGGCTGAGGACTTTGTACATTGTTTTGTTTTTTTTTTTTTTTGGTTTTGTCTTTTTTTAATAGTCATTCCAAGTATCCATGAAATAAGTGGTTACAGGAAGTCCCTCACCCTCCCAAAAGCCACCCCCACTCCTAAGAGGAGGATGGTCGCGTCCATGCCCTGAGTCCACCCCGGGGAAGGTGACAGCATTGCTTCTGTGTAAATTATGTACTGCAAAAATTTTTTTAAATCTTCCGCCTTAATACTTCATTTTTGTTTTTAATTTCTGAATGGCCCAGGTCTGAGGCCTCCCTTTTTTTTGTCCCCCCAACTTGATGTATGAAGGCTTTGGTCTCCCTGGGAGGGGGTTGAGGTGTTGAGGCAGCCAGGGCTGGCCTGTACACTGACTTGAGACCAATAAAAGTGCACACCTTACCTTACACAAAC

Mouse Gapdh mRNA:

>gi|126012538|ref|NM_008084.2| Mus musculus glyceraldehyde-3-phosphate dehydrogenase (Gapdh), mRNA:

AGAGACGGCCGCATCTTCTTGTGCAGTGCCAGCCTCGTCCCGTAGACAAAATGGTGAAGGTCGGTGTGAACGGATTTGGCCGTATTGGGCGCCTGGTCACCAGGGCTGCCATTTGCAGTGGCAAAGTGGAGATTGTTGCCATCAACGACCCCTTCATTGACCTCAACTACATGGTCTACATGTTCCAGTATGACTCCACTCACGGCAAATTCAACGGCACAGTCAAGGCCGAGAATGGGAAGCTTGTCATCAACGGGAAGCCCATCACCATCTTCCAGGAGCGAGACCCCACTAACATCAAATGGGGTGAGGCCGGTGCTGAGTATGTCGTGGAGTCTACTGGTGTCTTCACCACCATGGAGAAGGCCGGGGCCCACTTGAAGGGTGGAGCCAAAAGGGTCATCATCTCCGCCCCTTCTGCCGATGCCCCCATGTTTGTGATGGGTGTGAACCACGAGAAATATGACAACTCACTCAAGATTGTCAGCAATGCATCCTGCACCACCAACTGCTTAGCCCCCCTGGCCAAGGTCATCCATGACAACTTTGGCATTGTGGAAGGGCTCATGACCACAGTCCATGCCATCACTGCCACCCAGAAGACTGTGGATGGCCCCTCTGGAAAGCTGTGGCGTGATGGCCGTGGGGCTGCCCAGAACATCATCCCTGCATCCACTGGTGCTGCCAAGGCTGTGGGCAAGGTCATCCCAGAGCTGAACGGGAAGCTCACTGGCATGGCCTTCCGTGTTCCTACCCCCAATGTGTCCGTCGTGGATCTGACGTGCCGCCTGGAGAAACCTGCCAAGTATGATGACATCAAGAAGGTGGTGAAGCAGGCATCTGAGGGCCCACTGAAGGGCATCTTGGGCTACACTGAGGACCAGGTTGTCTCCTGCGACTTCAACAGCAACTCCCACTCTTCCACCTTCGATGCCGGGGCTGGCATTGCTCTCAATGACAACTTTGTCAAGCTCATTTCCTGGTATGACAATGAATACGGCTACAGCAACAGGGTGGTGGACCTCATGGCCTACATGGCCTCCAAGGAGTAAGAAACCCTGGACCACCCACCCCAGCAAGGACACTGAGCAAGAGAGGCCCTATCCCAACTCGGCCCCCAACACTGAGCATCTCCCTCACAATTTCCATCCCAGACCCCCATAATAACAGGAGGGGCCTAGGGAGCCCTCCCTACTCTCTTGAATACCATCAATAAAGTTCGCTGCACCCACAAAAAAAAAAAAAAAAAAAAAA

Mouse Hprt mRNA:

>gi|96975137|ref|NM_013556.2| Mus musculus hypoxanthine guanine phosphoribosyl transferase (Hprt), mRNA:

GGAGCCTGGCCGGCAGCGTTTCTGAGCCATTGCTGAGGCGGCGAGGGAGAGCGTTGGGCTTACCTCACTGCTTTCCGGAGCGGTAGCACCTCCTCCGCCGGCTTCCTCCTCAGACCGCTTTTTGCCGCGAGCCGACCGGTCCCGTCATGCCGACCCGCAGTCCCAGCGTCGTGATTAGCGATGATGAACCAGGTTATGACCTAGATTTGTTTTGTATACCTAATCATTATGCCGAGGATTTGGAAAAAGTGTTTATTCCTCATGGACTGATTATGGACAGGACTGAAAGACTTGCTCGAGATGTCATGAAGGAGATGGGAGGCCATCACATTGTGGCCCTCTGTGTGCTCAAGGGGGGCTATAAGTTCTTTGCTGACCTGCTGGATTACATTAAAGCACTGAATAGAAATAGTGATAGATCCATTCCTATGACTGTAGATTTTATCAGACTGAAGAGCTACTGTAATGATCAGTCAACGGGGGACATAAAAGTTATTGGTGGAGATGATCTCTCAACTTTAACTGGAAAGAATGTCTTGATTGTTGAAGATATAATTGACACTGGTAAAACAATGCAAACTTTGCTTTCCCTGGTTAAGCAGTACAGCCCCAAAATGGTTAAGGTTGCAAGCTTGCTGGTGAAAAGGACCTCTCGAAGTGTTGGATACAGGCCAGACTTTGTTGGATTTGAAATTCCAGACAAGTTTGTTGTTGGATATGCCCTTGACTATAATGAGTACTTCAGGGATTTGAATCACGTTTGTGTCATTAGTGAAACTGGAAAAGCCAAATACAAAGCCTAAGATGAGCGCAAGTTGAATCTGCAAATACGAGGAGTCCTGTTGATGTTGCCAGTAAAATTAGCAGGTGTTCTAGTCCTGTGGCCATCTGCCTAGTAAAGCTTTTTGCATGAACCTTCTATGAATGTTACTGTTTTATTTTTAGAAATGTCAGTTGCTGCGTCCCCAGACTTTTGATTTGCACTATGAGCCTATAGGCCAGCCTACCCTCTGGTAGATTGTCGCTTATCTTGTAAGAAAAACAAATCTCTTAAATTACCACTTTTAAATAATAATACTGAGATTGTATCTGTAAGAAGGATTTAAAGAGAAGCTATATTAGTTTTTTAATTGGTATTTTAATTTTTATATATTCAGGAGAGAAAGATGTGATTGATATTGTTAATTTAGACGAGTCTGAAGCTCTCGATTTCCTATCAGTAACAGCATCTAAGAGGTTTTGCTCAGTGGAATAAACATGTTTCAGCAGTGTTGGCTGTATTTTCCCACTTTCAGTAAATCGTTGTCAACAGTTCCTTTTAAATGCAAATAAATAAATTCTAAAAATTC

Figure S1.
