# Supplementary material for: Pseudogenes as Weaknesses of ACTB (Actb) and GAPDH (Gapdh) Used as Reference Genes in Reverse Transcription and Polymerase Chain Reactions
Source: PLoS One. 2012 Aug 22;7(8):e41659. doi: 10.1371/journal.pone.0041659 (PMC3425558; doi:10.1371/journal.pone.0041659)
Supplement: Figure S3 — Putative PGs of the Actb identified by Blat search using the Actb mRNA sequence. The top sequence that has 100% identity to the bait is the authentic Actb gene on mouse chromosome 5. The six genomic DNA fragments in the red box that have the highest scores to the bait were used in the alignment with the bait sequence shown in figure 4. (DOC) [file pone.0041659.s003.doc]

Figure S3:
