# Supplementary material for: Pseudogenes as Weaknesses of ACTB (Actb) and GAPDH (Gapdh) Used as Reference Genes in Reverse Transcription and Polymerase Chain Reactions
Source: PLoS One. 2012 Aug 22;7(8):e41659. doi: 10.1371/journal.pone.0041659 (PMC3425558; doi:10.1371/journal.pone.0041659)
Supplement: Figure S4 — Putative PGs of the GAPDH identified by Blat search using the GAPDH mRNA sequence (after deletion of the poly-A tail). The top sequence that has 100% identity to the bait is the authentic GAPDH gene on human chromosome 12. The seven genomic DNA fragments in the red box that have the highest scores to the bait were used in the alignment with the bait sequence shown in figure 5. (DOC) [file pone.0041659.s004.doc]

Figure S4:
