# Supplementary material for: Pseudogenes as Weaknesses of ACTB (Actb) and GAPDH (Gapdh) Used as Reference Genes in Reverse Transcription and Polymerase Chain Reactions
Source: PLoS One. 2012 Aug 22;7(8):e41659. doi: 10.1371/journal.pone.0041659 (PMC3425558; doi:10.1371/journal.pone.0041659)
Supplement: Figure S5 — Putative PGs of the Gapdh identified by Blat search using the Gapdh mRNA sequence (after deletion of the poly-A tail). The top sequence that has 100% identity to the bait is the authentic Gapdh gene on the mouse chromosome 7. The seven genomic DNA fragments in the red box that have the highest scores to the bait were used in the alignment with the bait sequence shown in figure 6. (DOC) [file pone.0041659.s005.doc]

(continuing to the next page)

Figure S5
